# Supplementary material for: Diagnosis and Improvement of Combustion Characteristics of Methanol Miniature Reciprocating Piston Internal Combustion Engine
Source: Micromachines (Basel). 2020 Jan 16;11(1):96. doi: 10.3390/mi11010096 (PMC7020190; doi:10.3390/mi11010096)
Supplement: Supplementary file 1 [file micromachines-11-00096-s001.zip › micromachines-675109 suppl. for final.docx]

Diagnosis and Improvement of Combustion Characteristics of Methanol Miniature Reciprocating Piston Internal Combustion Engine

Gangzhi Tang ^1,^*, Shuaibin Wang ^1^, Li Zhang ^2^ and Huichao Shang ^2^

**Table S1.** Methanol-nitromethane mixed fuel combustion chemical reaction mechanism.

| **NO.** | **Reaction** | ***A_r_*** | ***β_r_*** | ***E_r_*** |
| --- | --- | --- | --- | --- |
| 1 | H+O_2_=O+OH | 3.55E+15 | -0.40 | 1.66E+04 |
| 2 | O+H_2_=H+OH | 5.08E+04 | 2.70 | 6.29E+03 |
| 3 | H_2_+OH=H_2_O+H | 2.16E+08 | 1.50 | 3.43E+03 |
| 4 | O+H_2_O=OH+OH | 2.97E+06 | 2.00 | 1.34E+04 |
| 5 | H_2_+M=H+H+M | 4.58E+19 | -1.40 | 1.04E+05 |
| 6 | O+O+M=O_2_+M | 6.16E+15 | -0.50 | 0.00 |
| 7 | O+H+M=OH+M | 4.71E+18 | -1.00 | 0.00 |
| 8 | H+OH+M=H_2_O+M | 3.80E+12 | -2.00 | 0.00 |
| 9 | H+O_2_(+M)=HO_2_(+M) | 1.48E+12 | 0.60 | 0.00 |
| 10 | HO_2_+H=H_2_+O_2_ | 1.66E+13 | 0.00 | 823.00 |
| 11 | HO_2_+H=OH+OH | 7.08E+13 | 0.00 | 295.00 |
| 12 | HO_2_+O=O_2_+OH | 3.25E+13 | 0.00 | 0.00 |
| 13 | HO_2_+OH=H_2_O+O_2_ | 2.89E+13 | 0.00 | -497.00 |
| 14 | HO_2_+HO_2_=H_2_O_2_+O_2_ | 4.20E+14 | 0.00 | 12000 |
| 15 | H_2_O_2_(+M)=OH+OH(+M) | 2.95E+14 | 0.00 | 48400.00 |
| 16 | H_2_O_2_+H=H_2_O+OH | 2.41E+13 | 0.00 | 3970.00 |
| 17 | H_2_O_2_+H=HO_2_+H_2_ | 4.82E+13 | 0.00 | 7.95E+03 |
| 18 | H_2_O_2_+O=OH+HO_2_ | 9.55E+06 | 2.00 | 3.97E+03 |
| 19 | H_2_O_2_+OH=HO_2_+H_2_O | 1.00E+12 | 0.00 | 0.00 |
| 20 | CO+O(+M)=CO_2_(+M) | 1.8E+10 | 0.00 | 2.38E+03 |
| 21 | CO+O_2_=CO_2_+O | 2.53E+12 | 0.00 | 4.77E+04 |
| 22 | CO+HO_2_=CO_2_+OH | 3.01E+13 | 0.00 | 2.30E+04 |
| 23 | CO+OH=CO_2_+H | 2.23E+05 | 1.90 | -1.16E+03 |
| 24 | HCO+M=H+CO+M | 4.75E+11 | 0.7 | 1.49E+04 |
| 25 | HCO+O_2_=CO+HO_2_ | 7.58E+12 | 0.00 | 4.10E+02 |
| 26 | HCO+H=CO+H_2_ | 7.23E+13 | 0.00 | 0.00 |
| 27 | HCO+O=CO+OH | 3.02E+13 | 0.00 | 0.00 |
| 28 | HCO+OH=CO+H_2_O | 3.02E+13 | 0.00 | 0.00 |
| 29 | HCO+O=CO_2_+H | 3.00E+13 | 0.00 | 0.00 |
| 30 | HCO+HO_2_=CO_2_+OH+H | 3.00E+13 | 0.00 | 0.00 |
| 31 | HCO+HCO=H_2_+CO+CO | 3.00E+12 | 0.00 | 0.00 |
| 32 | HCO+CH_3_=CO+CH_4_ | 1.20E+14 | 0.00 | 0.00 |
| 33 | HCO+HCO=CH_2_O+CO | 3.00E+13 | 0.00 | 0.00 |
| 34 | CH_2_O+M=HCO+H+M | 3.30E+39 | -6.30 | 9.99E+04 |
| 35 | CH_2_O+M=CO+H_2_+M | 3.10E+45 | -8.00 | 9.75E+04 |
| 36 | CH_2_O+H=HCO+H_2_ | 5.74E+07 | 1.90 | 2.75E+03 |
| 37 | CH_2_O+O=HCO+OH | 1.81E+13 | 0.00 | 3.08E+03 |
| 38 | CH_2_O+OH=HCO+H_2_O | 3.43E+09 | 1.20 | -4.47E+02 |
| 39 | CH_2_O+O_2_=HCO+HO_2_ | 1.23E+06 | 3.00 | 5.20E+04 |
| 40 | CH_2_O+HO_2_=HCO+H_2_O_2_ | 4.11E+04 | 2.50 | 1.02E+04 |
| 41 | CH_2_O+CH_3_=HCO+CH_4_ | 3.64E-06 | 5.40 | 9.98E+02 |
| 42 | CH_3_+O=CH_2_O+H | 8.43E+13 | 0.00 | 0.00 |
| 43 | CH_3+_O_2_=CH_3_O+O | 1.99E+18 | -1.60 | 2.92E+04 |
| 44 | CH_3_+O_2_=CH_2_O+OH | 3.74E+11 | 0.00 | 1.46E+04 |
| 45 | CH_3_+HO_2_=CH_3_O+OH | 2.41E+10 | 0.80 | -2.33E+03 |
| 46 | CH_3_+H(+M)=CH_4_(+M) | 1.27E+16 | -0.60 | 3.83E+02 |
| 47 | CH_4_+H=CH_3_+H_2_ | 5.47E+07 | 2.00 | 1.12E+04 |
| 48 | CH_4_+O=CH_3_+OH | 3.15E+12 | 0.50 | 1.03E+04 |
| 49 | CH_4_+OH=CH_3_+H_2_O | 5.72E+06 | 2.00 | 2.64E+03 |
| 50 | CH_3_+HO_2_=CH_4_+O_2_ | 3.16E+12 | 0.00 | 0.00 |
| 51 | CH_4_+HO_2_=CH_3_+H_2_O_2_ | 1.81E+11 | 0.00 | 1.86E+04 |
| 52 | CH_2_OH+M=CH_2_O+H+M | 1.00E+14 | 0.00 | 2.51E+04 |
| 53 | CH_2_OH+H=CH_2_O+H_2_ | 6.00E+12 | 0.00 | 0.00 |
| 54 | CH_2_OH+H=CH3+OH | 9.64E+13 | 0.00 | 0.00 |
| 55 | CH_2_OH+O=CH_2_O+OH | 4.20E+13 | 0.00 | 0.00 |
| 56 | CH_2_OH+OH=CH_2_O+H_2_O | 2.40E+13 | 0.00 | 0.00 |
| 57 | CH_2_OH+O_2_=CH_2_O+HO_2_ | 2.41E+14 | 0.00 | 5.02E+03 |
| 58 | CH_2_OH+HO_2_=CH_2_O+H_2_O_2_ | 1.20E+13 | 0.00 | 0.00 |
| 59 | CH_2_OH+HCO=CH_3_OH+CO | 1.00E+13 | 0.00 | 0.00 |
| 60 | CH_2_OH+HCO=CH_2_O+CH_2_O | 1.50E+13 | 0.00 | 0.00 |
| 61 | CH_2_OH+CH_2_OH=CH_3_OH+CH_2_O | 3.00E+12 | 0.00 | 0.00 |
| 62 | CH_2_OH+CH_3_O=CH_3_OH+CH2O | 2.40E+13 | 0.00 | 0.00 |
| 63 | CH_3_O+M=CH_2_O+H+M | 8.30E+17 | -1.20 | 1.55E+04 |
| 64 | CH_3_O+H=CH_3_+OH | 3.20E+13 | 0.00 | 0.00 |
| 65 | CH_3_O+O=CH_2_O+OH | 6.00E+12 | 0.00 | 0.00 |
| 66 | CH_3_O+OH=CH_2_O+H_2_O | 1.80E+13 | 0.00 | 0.00 |
| 67 | CH_3_O+O_2_=CH_2_O+HO_2_ | 9.03E+13 | 0.00 | 1.20E+04 |
| 68 | CH_3_O+HO_2_=CH_2_O+H_2_O_2_ | 3.00E+11 | 0.00 | 0.00 |
| 69 | CH_3_O+CO=CH_3_+CO_2_ | 1.60E+13 | 0.00 | 1.18E+04 |
| 70 | CH_3_O+HCO=CH_3_OH+CO | 9.00E+13 | 0.00 | 0.00 |
| 71 | CH_3_O+CH_3_O=CH_3_OH+CH_2_O | 6.00E+13 | 0.00 | 0.00 |
| 72 | OH+CH_3_(+M)=CH_3_OH(+M) | 2.79E+18 | -1.40 | 1.33E+03 |
| 73 | H+CH_2_OH(+M)=CH_3_OH(+M) | 1.06E+12 | 0.50 | 8.60E+01 |
| 74 | H+CH_3_O(+M)=CH_3_OH(+M) | 2.43E+12 | 0.50 | 5.00E+01 |
| 75 | CH_3_OH+H=CH_2_OH+H_2_ | 3.20E+13 | 0.00 | 6.10E+03 |
| 76 | CH_3_OH+H=CH_3_O+H_2_ | 8.00E+12 | 0.00 | 6.10E+03 |
| 77 | CH_3_OH+O=CH_2_OH+OH | 3.88E+05 | 2.50 | 3.08E+03 |
| 78 | CH_3_OH+OH=CH_3_O+H_2_O | 1.00E+06 | 2.10 | 4.97E+02 |
| 79 | CH_3_OH+OH=CH_2_OH+H_2_O | 7.10E+06 | 1.80 | -5.96E+02 |
| 80 | CH_3_OH+O_2_=CH_2_OH+HO_2_ | 2.05E+13 | 0.00 | 4.49E+04 |
| 81 | CH_3_OH+HCO=CH_2_OH+CH_2_O | 9.64E+03 | 2.90 | 1.31E+04 |
| 82 | CH_3_OH+HO_2_=CH_2_OH+H_2_O_2_ | 3.98E+13 | 0.00 | 1.94E+04 |
| 83 | CH_3_OH+CH_3_=CH_2_OH+CH_4_ | 3.19E+01 | 3.20 | 7.17E+03 |
| 84 | CH_3_O+CH3OH=CH_3_OH+CH_2_OH | 3.00E+11 | 0.00 | 4.06E+03 |
| 85 | CH_3_NO_2_(+M)=CH_3_+NO_2_(+M) | 1.80E+16 | 0.0 | 58500.0 |
| 86 | CH_3_NO_2_+H=HONO+CH_3_ | 3.30E+12 | 0.0 | 3730.0 |
| 87 | CH_3_NO_2_+H=CH_3_NO+OH | 1.40E+12 | 0.0 | 3730.0 |
| 88 | CH_3_NO_2_+H=CH_2_NO_2_+H_2_ | 5.40E+02 | 3.5 | 5200.0 |
| 89 | CH_3_NO_2_+O=CH_2_NO_2_+OH | 1.50E+13 | 0.0 | 5350.0 |
| 90 | CH_3_NO_2_+OH=CH_2_NO_2_+H_2_O | 5.00E+05 | 2.0 | 1000.0 |
| 91 | CH_3_NO_2_+OH=CH_3_OH+NO_2_ | 2.0E+10 | 0.00 | -1000.0 |
| 92 | CH_3_NO_2_+HO_2_=CH_2_NO_2_+H_2_O_2_ | 3.00E+12 | 0.0 | 23000.0 |
| 93 | CH_3_NO_2_+O_2_=CH_2_NO_2_+HO_2_ | 2.00E+13 | 0.0 | 57000.0 |
| 94 | CH_3_NO_2_+CH_3_=CH_2_NO_2_+CH_4_ | 5.50E-01 | 4.0 | 8300.0 |
| 95 | CH_3_NO_2_+CH_3_O=CH_2_NO_2_+CH_3_OH | 3.00E+11 | 0.0 | 7000.0 |
| 96 | CH_3_NO_2_+NO_2_=CH_2_NO_2_+HONO | 3.00E+11 | 0.0 | 32000.0 |
| 97 | CH_3_+NO(+M)=CH_3_NO(+M) | 9.00E+12 | 0.0 | 119.0 |
| 98 | CH_3_NO+H=CH_2_NO+H_2_ | 4.40E+08 | 1.5 | 378.0 |
| 99 | CH_3_NO+O=CH_2_NO+OH | 3.30E+08 | 1.5 | 3616.0 |
| 100 | CH_3_NO+OH=CH_2_NO+H_2_O | 3.60E+06 | 2.0 | -1192.0 |
| 101 | CH_3_NO+CH_3_=CH_2_NO+CH_4_ | 7.90E+05 | 1.9 | 5415.0 |
| 102 | CH_3_NO+NH_2_=CH_2_NO+NH_3_ | 2.80E+06 | 1.9 | 1073.0 |
| 103 | CH_3_NO+H=CH_3_+HNO | 1.80E+13 | 0.0 | 2782.0 |
| 104 | CH_3_NO+O=CH_3_+NO_2_ | 1.70E+06 | 2.1 | 0.0 |
| 105 | CH_3_NO+OH=CH_3_+HONO | 2.50E+12 | 0.0 | 994.0 |
| 107 | CH_2_NO_2_+H=CH_3_+NO_2_ | 5.00E+13 | 0.0 | 0.0 |
| 108 | CH_2_NO_2_+O=CH_2_O+NO_2_ | 5.00E+13 | 0.0 | 0.0 |
| 109 | CH_2_NO_2_+OH=CH_2_OH+NO_2_ | 1.00E+13 | 0.0 | 0.0 |
| 110 | CH_2_NO_2_+OH=CH_2_O+HONO | 1.00E+13 | 0.0 | 0.0 |
| 111 | CH_2_NO=HNCO+H | 6.90E+41 | -9.3 | 51702.0 |
| 112 | CH_2_NO+H=CH_3_+NO | 4.00E+13 | 0.0 | 0.0 |
| 113 | CH_2_NO+O=CH_2_O+NO | 7.00E+13 | 0.0 | 0.0 |
| 114 | CH_2_NO+OH=CH_2_OH+NO | 4.00E+13 | 0.0 | 0.0 |
| 115 | CH_2_NO+O_2_=CH_2_O+NO_2_ | 1.10E+23 | -3.3 | 3895.0 |
| 116 | CH_3_+NO_2_=CH_3_ONO | 5.00E+11 | 0 | 0 |
| 117 | CH_3_+NO_2_=CH_3_O+NO | 4.00E+13 | -0.2 | 0 |
| 118 | CH_3_+OH=CH_2_O+H_2_ | 8.00E+12 | 0 | 0 |
| 119 | CH_3_+NO=HCN+H_2_O | 1.50E-01 | 3.5 | 3950.0 |
| 120 | CH_3_+HNO=CH_4_+NO | 2.30E+14 | 0 | 8400.0 |
| 121 | CH_3_ONO+H=CH_3_OH+NO | 1.20E+11 | 0.0 | 1900.0 |
| 122 | CH_3_ONO+H=CH_2_O+H_2_+NO | 1.40E+11 | 0.0 | 1900.0 |
| 123 | CH_3_ONO+O=CH_3_O+NO_2_ | 1.40E+13 | 0.0 | 5210.0 |
| 124 | CH_3_ONO+OH=CH_3_OH+NO_2_ | 6.00E+13 | 0.0 | 3505.0 |
| 125 | HNCO(+M)=CO+NH(+M) | 6.00E+13 | 0.0 | 99800.0 |
| 126 | HNCO+H=NH_2_+CO | 2.2E+07 | 1.7 | 3800 |
| 127 | HNCO+O=NCO+OH | 2.20E+06 | 2.1 | 11430.0 |
| 128 | HNCO+O=NH+CO_2_ | 9.60E+07 | 1.4 | 8520.0 |
| 129 | HNCO+O=HNO+CO | 1.50E+08 | 1.6 | 44012.0 |
| 130 | HNCO+OH=NCO+H_2_O | 6.38E+05 | 2 | 2563.0 |
| 131 | HNCO+HO_2_=NCO+H_2_O_2_ | 3.00E+11 | 0.0 | 22000.0 |
| 132 | HNCO+O_2_=HNO+CO_2_ | 1.00E+12 | 0.0 | 35000.0 |
| 133 | HNCO+NH=NH_2_+NCO | 3.00E+13 | 0.0 | 23700.0 |
| 134 | HCN(+M)=H+CN(+M) | 8.30E+17 | -0.9 | 123800.0 |
| 135 | HCN+O=NCO+H | 1.40E+04 | 2.6 | 4980.0 |
| 136 | HCN+O=CN+OH | 2.70E+9 | 1.6 | 29200.0 |
| 137 | HCN+O=NH+CO | 3.50E+03 | 2.6 | 4980.0 |
| 138 | HCN+OH=CN+H_2_O | 3.90E+06 | 1.8 | 10300.0 |
| 139 | HCN+OH=HOCN+H | 5.90E+04 | 2.4 | 12500.0 |
| 140 | HCN+OH=HNCO+H | 2.00E-03 | 4.0 | 1000.0 |
| 141 | HCN+OH=NH_2_+CO | 7.80E-04 | 4.0 | 4000.0 |
| 142 | HCN+O_2_=CN+HO_2_ | 3.00E+13 | 0.0 | 75100.0 |
| 143 | HOCN+H=HNCO+H | 2.00E+07 | 2.0 | 2000.0 |
| 144 | HOCN+H=H_2_+NCO | 2.40E+08 | 1.5 | 6617.0 |
| 145 | HOCN+O=OH+NCO | 1.50E+04 | 2.6 | 4000.0 |
| 146 | HOCN+OH=H_2_O+NCO | 6.40E+05 | 2.0 | 2560.0 |
| 147 | CN+O=CO+N | 1.80E+13 | 0 | 0 |
| 148 | CN+OH=NCO+H | 4.22E+13 | 0 | 0.0 |
| 149 | CN+O_2_=NCO+O | 7.50E+12 | 0.0 | -389.0 |
| 150 | CN+O_2_=NO+CO | 2.80E+17 | -2.0 | 0.0 |
| 151 | CN+NO=NCO+N | 9.60E+13 | 0.0 | 42100.0 |
| 152 | CN+NO_2_=NCO+NO | 5.30E+15 | -0.8 | 344.0 |
| 153 | CN+NO_2_=CO+N_2_O | 4.90E+14 | -0.8 | 344.0 |
| 154 | CN+HNO=HCN+NO | 1.80E+13 | 0.0 | 0.0 |
| 155 | CN+HONO=HCN+NO_2_ | 1.20E+13 | 0.0 | 0.0 |
| 156 | NCO+M=N+CO+M | 3.10E+16 | -0.5 | 48300.0 |
| 157 | NCO+H=CO+NH | 5.36E+13 | 0.0 | 0 |
| 158 | NCO+H_2_=HNCO+H | 7.6E+02 | 3.0 | 4000 |
| 159 | NCO+O=NO+CO | 2.00E+13 | 0 | 0.0 |
| 160 | NCO+OH=H+CO+NO | 8.30E+12 | 0 | 0 |
| 161 | NCO+HO_2_=HNCO+O_2_ | 2.00E+13 | 0.0 | 0.0 |
| 162 | NCO+O_2_=NO+CO_2_ | 2.00E+12 | 0.0 | 20000.0 |
| 163 | NCO+NO=N_2_O+CO | 6.2E+17 | -1.7 | 763.0 |
| 164 | NCO+NO=N_2_+CO_2_ | 7.80E+17 | -1.7 | 763.0 |
| 165 | NCO+NO_2_=CO+NO+NO | 1.39E+13 | 0.0 | 0.0 |
| 166 | NCO+NO_2_=CO_2_+N_2_O | 4.17E+12 | 0.0 | 0.0 |
| 167 | NH_2_+H=NH+H_2_ | 6.92E+13 | 0 | 3650 |
| 168 | NH_2_+O=HNO+H | 6.60E+14 | -0.5 | 0.0 |
| 169 | NH_2_+O=NH+OH | 6.75E+12 | 0.0 | 0.0] |
| 170 | NH_2_+OH=NH+H_2_O | 4.00E+06 | 2.0 | 1000.0 |
| 171 | NH_2_+O_2_=HNO+OH | 1.78E+12 | 0 | 14900 |
| 172 | NH_2_+NO=N_2_+H_2_O | 1.3E+16 | -1.2 | 0 |
| 173 | NH_2_+NO_2_=N_2_O+H_2_O | 3.28E+18 | -2.2 | 0 |
| 174 | NH+O=NO+H | 5.5E+13 | 0.0 | 0.0 |
| 175 | NH+OH=HNO+H | 2.0E+13 | 0.0 | 0.0 |
| 176 | NH+O_2_=HNO+O | 3.89E+13 | 0 | 17890 |
| 177 | NH+O_2_=NO+OH | 7.6E+10 | 1.5 | 1530 |
| 178 | NH+NO=N_2_O+H | 2.90E+14 | -0.4 | 0.0 |
| 179 | NH+NO=N_2_+OH | 2.20E+13 | -0.2 | 0.0 |
| 180 | NH+NO_2_=N_2_O+OH | 1.00E+13 | 0.0 | 0.0 |
| 181 | NO+CH_2_OH=HNO+CH_2_O | 1.3E+12 | 0.00 | 0 |
| 182 | NO+CH_3_O=HNO+CH_2_O | 7.5E+12 | 0.00 | 2017 |
| 183 | NO+CH_3_O(+M)=CH_3_ONO(+M) | 6.0E+14 | -0.60 | 0 |
| 184 | NO+HCO=HNO+CO | 7.1E+12 | 0.00 | 0 |
| 185 | NO+H(+M)=HNO(+M) | 1.5E+15 | -0.41 | 0 |
| 186 | NO+O(+M)=NO_2_(+M) | 1.3E+15 | -0.75 | 0 |
| 187 | NO+OH(+M)=HONO(+M) | 1.1E+14 | -0.30 | 0 |
| 188 | NO+HO_2_=NO_2_+OH | 2.1E+12 | 0.00 | -500 |
| 189 | NO_2_+CH_3_OH=HONO+CH_2_OH | 1.5E+02 | 3.32 | 20035 |
| 190 | NO_2_+CH_3_OH=HNO_2_+CH_2_OH | 2.4E+03 | 2.90 | 27470 |
| 191 | NO_2_+CH_2_OH=HONO+CH_2_O | 5.0E+12 | 0.00 | 0 |
| 192 | NO_2_+CH_3_O=HONO+CH_2_O | 6.0E+12 | 0.00 | 2285 |
| 193 | NO_2_+CH_3_O(+M)=CH_3_ONO_2_(+M) | 2.2E+15 | -0.88 | 0 |
| 194 | NO_2_+CH_2_O=HONO+HCO | 1.4E-7 | 5.64 | 9220 |
| 195 | NO_2_+CH_2_O=HNO_2_+HCO | 1.1E-1 | 4.22 | 19850 |
| 196 | NO_2_+HCO=NO+CO_2_+H | 2.3E+13 | 0.00 | 0 |
| 197 | NO_2_+HCO=HONO+CO | 5.0E+12 | 0.00 | 0 |
| 198 | NO_2_+HCO=NO+CO+OH | 5.0E+12 | 0.00 | 0 |
| 199 | NO_2_+CO=NO+CO_2_ | 9.0E+13 | 0.00 | 33800 |
| 200 | NO_2_+H=HNO_2_ | 5.0E+19 | -3.00 | 0 |
| 201 | NO_2_+H=NO+OH | 1.3E+14 | 0.00 | 362 |
| 202 | NO_2_+H_2_=HONO+H | 1.3E+04 | 2.76 | 29770 |
| 203 | NO_2_+H_2_=HNO_2_+H | 2.4 | 3.73 | 32400 |
| 204 | NO_2_+O=NO+O_2_ | 1.1E+14 | -0.52 | 0 |
| 205 | NO_2_+HO_2_=HONO+O_2_ | 1.9 | 3.32 | 3044 |
| 206 | NO_2_+HO_2_=HNO_2_+O_2_ | 1.9E+01 | 3.26 | 4983 |
| 207 | NO_2_+NO_2_=NO+NO+O_2_ | 4.5E+12 | 0.00 | 27600 |
| 208 | HNO+CH_2_OH=NO+CH_3_OH | 3.0E+13 | 0.00 | 0 |
| 209 | HNO+CH_3_O=NO+CH_3_OH | 3.2E+13 | 0.00 | 0 |
| 210 | HNO+HCO=NO+CH_2_O | 5.8E-1 | 3.84 | 115 |
| 211 | HNO+H=NO+H_2_ | 4.4E+11 | 0.72 | 650 |
| 212 | HNO+O=NO+OH | 2.3E+13 | 0.00 | 0 |
| 213 | HNO+OH=NO+H_2_O | 1.3E+07 | 1.88 | -956 |
| 214 | HNO+O_2_=HO_2_+NO | 2.0E+13 | 0.00 | 16000 |
| 215 | HNO+NO_2_=HONO+NO | 4.4E+04 | 2.64 | 4040 |
| 216 | HONO+O=NO_2_+OH | 1.2E+13 | 0.00 | 5960 |
| 217 | HONO+OH=NO_2_+H_2_O | 1.7E+12 | 0.00 | -520 |
| 218 | HONO+HONO=NO+NO_2_+H_2_O | 3.5E-1 | 3.64 | 12140 |
| 219 | HNO_2_(+M)=HONO(+M) | 2.5E+14 | 0.00 | 32300 |
| 220 | HNO_2_+O=NO_2_+OH | 1.7E+08 | 1.50 | 2000 |
| 221 | HNO_2_+OH=NO_2_+H_2_O | 4.0E+13 | 0.00 | 0 |
| 222 | CH_3_ONO_2_+H=HONO+CH_3_O | 1.0E+12 | 0.00 | 0 |
| 223 | CH_3_ONO_2_+O=NO_3_+CH_3_O | 1.5E+13 | 0.00 | 5260 |
| 224 | N+NO=N_2_+O | 2.700E+13 | 0.000 | 355.00 |
| 225 | N+O_2_=NO+O | 9.000E+09 | 1.000 | 6500.00 |
| 226 | N+OH=NO+H | 3.360E+13 | 0.000 | 385.00 |
| 227 | HNO+NO=N_2_O+OH | 1.70E+13 | 0.0 | 29590.0 |
| 228 | HNO+HNO=H_2_O+N_2_O | 8.51E+08 | 0.0 | 3080.0 |
| 229 | N+NO_2_=N_2_O+O | 5.00E+12 | 0.0 | 0.0 |
| 230 | NH_2_+NO=N_2_O+H_2_ | 5.00E+13 | 0.0 | 24640.0 |
| 231 | N_2_O+O=2NO | 1.00E+14 | 0.0 | 28000.0 |
| 232 | N_2_O(+M)=N_2_+O(+M) | 7.91E+10 | 0.0 | 56020.0 |
| 233 | N_2_O+CO=N_2_+CO_2_ | 5.01E+13 | 0.0 | 44000.0 |
| 234 | N_2_O+H=N_2_+OH | 2.53E+10 | 0.0 | 4550.0 |
| 235 | N_2_O+O=O_2_+N_2_ | 1.00E+14 | 0.0 | 28000.0 |
| 236 | NCO+N_2_O=N_2_+NO+CO | 9.03E+13 | 0.0 | 27820.0 |
| 237 | NNH+NO=N_2_+HNO | 5.00E+13 | 0.0 | 0.0 |
| 238 | NH_2_+NO=NNH+OH | 2.80E+13 | -0.6 | 0.0 |
| 239 | NNH+M=N_2_+H+M | 1.00E+14 | 0.0 | 3000.0 |
| 240 | NNH+OH=N_2_+H_2_O | 5.00E+13 | 0.0 | 0.0 |

*A_r_*: Pre-exponential；*β_r_*: Temperature exponent；*E_r_*: Reaction activation energy.
